# Mesalamine as a Suspected Cause of Drug-Induced Pancreatitis: A Case Report and Review of Diagnostic Considerations

**DOI:** 10.7759/cureus.92923

**Published:** 2025-09-22

**Authors:** Elias Arellano Villanueva, Miguel Lopez, Alhasan Asaad, Jose E Campo Maldonado

**Affiliations:** 1 Department of Internal Medicine, The University of Texas Rio Grande Valley School of Medicine, Edinburg, USA; 2 Department of Internal Medicine, Valley Baptist Medical Center/The University of Texas Rio Grande Valley, Edinburg, USA; 3 Department of Internal Medicine, Division of Infectious Disease, The University of Texas Rio Grande Valley School of Medicine, Edinburg, USA

**Keywords:** 5-aminosalicylic acid, acute pancreatitis, adverse drug reaction, chronic kidney disease, drug-induced pancreatitis, inflammatory bowel disease, mesalamine, pancreatic inflammation, polypharmacy

## Abstract

Acute pancreatitis (AP) is a common cause of gastrointestinal-related hospitalizations in the United States. It is characterized by pancreatic inflammation and necrosis, with diagnosis based on elevated pancreatic enzymes, characteristic imaging, and epigastric pain radiating to the back. While gallstones and alcohol are the predominant etiologies, drug-induced pancreatitis (DIP) accounts for up to 5% of cases and remains a diagnostic challenge. Over 500 medications, including mesalamine, have been implicated. Proposed mechanisms of DIP include pancreatic duct obstruction, toxic metabolite accumulation, hypersensitivity reactions, and localized angioedema. Prompt recognition and withdrawal of the offending agent are critical to prevent complications.

We report a 53-year-old female with multiple comorbidities, including chronic kidney disease and type 2 diabetes, who presented with persistent upper abdominal pain, nausea, and vomiting. Imaging revealed an enlarged pancreas with peripancreatic inflammation, consistent with AP, despite normal serum lipase levels. Laboratory workup excluded common etiologies such as gallstones, alcohol use, and hypertriglyceridemia. Given the temporal association, mesalamine was identified as the likely offending agent and discontinued, resulting in symptom resolution. The patient was managed with supportive care and discharged in stable condition.

DIP is a rare but important differential in AP, particularly in patients with complex comorbidities and polypharmacy. This case illustrates the diagnostic challenges of DIP and underscores the importance of thorough medication review and early withdrawal of the offending agent to ensure favorable outcomes. Clinician vigilance is essential in recognizing less common causes of pancreatitis, such as mesalamine-induced DIP.

## Introduction

Acute pancreatitis (AP) is the leading cause of gastrointestinal-related hospitalizations in the United States, accounting for approximately 300,000 emergency department (ED) visits per year [1]. AP is characterized by pancreatic and peripancreatic inflammation with fat necrosis [1]. The clinical diagnosis of AP requires the presence of at least two of the following three criteria: serum amylase and lipase levels elevated more than three times the upper limit of normal; mild to severe epigastric abdominal pain (often radiating to the back); and typical imaging features as found on computed tomography (CT) or magnetic resonance imaging [2]. The most common causes of AP in the United States are gallstones (35-40% of cases), alcohol use (30%) [3], idiopathic etiologies (10-30%), post-endoscopic retrograde cholangiopancreatography (ERCP) (3-5%), and medications (0.1%-5%) [4].

Drug-induced pancreatitis (DIP) is an uncommon but important cause of AP, with a prevalence of up to 5% [5]. Diagnosing DIP requires first confirming AP through elevated serum lipase or amylase levels, followed by systematic exclusion of more common causes such as gallstones, alcohol use, and hypertriglyceridemia through clinical history, imaging, and laboratory evaluation [6]. Drug classes most frequently associated with DIP include antiepileptic agents (e.g., valproic acid), antimicrobial agents (e.g., tetracyclines, metronidazole, sulfonamides), and immunosuppressive/anti-inflammatory agents (e.g., azathioprine, corticosteroids, 5-aminosalicylic acid derivatives) [6].

Common culprits include acetaminophen, metronidazole, trimethoprim-sulfamethoxazole, proton pump inhibitors (PPIs), valproate, tigecycline, and doxycycline [4]. Clinical presentation can range from mild to severe, with potential fatal outcomes [6]. Potential mechanisms for drug-induced AP include pancreatic duct constriction, cytotoxic and metabolic effects, toxic metabolite accumulation, hypersensitivity reactions, and localized angioedema or arteriolar thrombosis [6]. Additionally, drug-induced hypertriglyceridemia and chronic hypercalcemia may contribute, as these are established risk factors for AP [1,4].

Management of DIP primarily involves prompt withdrawal of the offending agent and supportive care [6]. However, identifying the causative drug can be particularly challenging in patients on multi-drug regimens, such as those undergoing cancer treatment, or with multiple comorbid conditions [7].

In this case report, we present a 53-year-old female with suspected DIP in the context of multiple comorbidities, including chronic kidney disease (CKD) and type 2 diabetes, highlighting the diagnostic challenges and clinical management considerations of DIP in patients with complex medication regimens.

## Case presentation

A 53-year-old female with a history of hypothyroidism, type 2 diabetes, atrial fibrillation, hypertension, dyslipidemia, CKD stage 3, and erosive gastritis presented to the ED with persistent upper abdominal pain rated at eight out of 10, nausea, and non-bloody vomiting. On admission, blood pressure was 160/69 mmHg. Otherwise, her vital signs were within normal limits.

**Figure 1:**
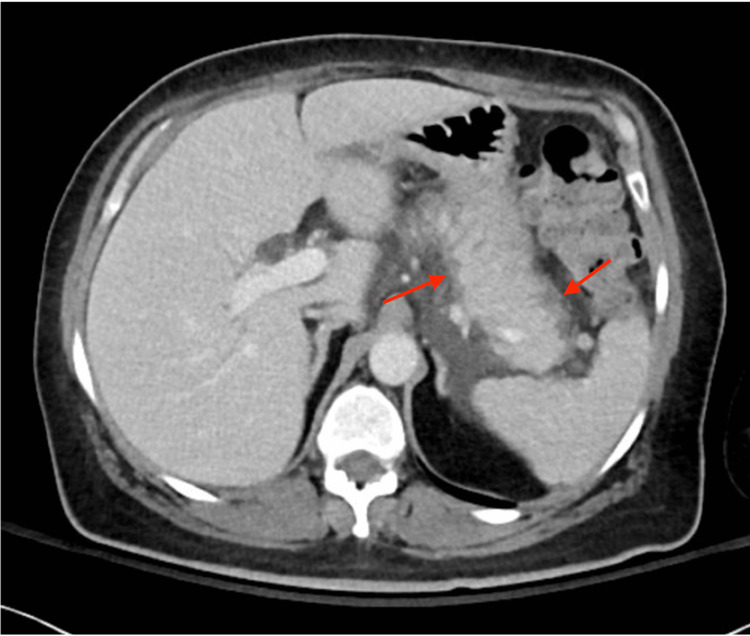
Axial abdominal CT showing diffuse enlargement of the pancreatic body and tail with peripancreatic fat stranding and no evidence of pancreatic necrosis Red arrows indicate peripancreatic fat stranding, a hallmark radiologic feature of acute pancreatitis, reflecting inflammation extending into the surrounding fat.

A CT scan of the abdomen and pelvis revealed an enlarged pancreas with peripancreatic fluid and fat stranding, consistent with AP (Figure 1).

Serum lipase levels, however, were within normal limits. Laboratory evaluation demonstrated leukocytosis (WBC 11.1 × 10⁹/L), mild hyperglycemia (glucose 151 mg/dL), and elevated creatinine (2.51 mg/dL, slightly elevated above her baseline of 2.33 mg/dL), total protein 7.4 g/dL, and urinalysis revealing trace protein and glucosuria without evidence of infection (Table 1).

**Table 1:** Hematological findings of the patient during the hospital stay ALP: alkaline phosphatase; ALT: alanine transaminase; AST: aspartate aminotransferase; CO₂: bicarbonate; eGFR CKD-EPI: estimated glomerular filtration rate (Chronic Kidney Disease Epidemiology Collaboration formula); HCT: hematocrit; MCH: mean corpuscular hemoglobin; MCHC: mean corpuscular hemoglobin concentration; MCV: mean corpuscular volume; MPV: mean platelet volume; TSH: thyroid-stimulating hormone. (L) indicates a value below the reference range; (H) indicates a value above the reference range. All values are in SI (International System of Units) units unless otherwise specified. Reference ranges are stratified by sex where applicable.

Laboratory Studies	Reference Range	Day 1	Day 2	Day 4	Day 5	Day 6
Erythrocytes (×10^12^/L)	4.7-6.1 (M); 4.2-5.4 (F)	3.35 (L)	3.55 (L)	3.66 (L)	3.40 (L)	3.24 (L)
Hemoglobin (g/L)	135-175 (M); 120-160 (F)	9.7 (L)	10.1 (L)	10.5 (L)	9.8 (L)	9.4 (L)
Hematocrit (%)	41-53 (M); 36-46 (F)	30.8 (L)	32.0 (L)	32.9 (L)	30.7 (L)	28.9 (L)
MCV (fL)	80-100	91.9	90.1	89.9	90.3	89.2
MCH (pg)	27-33	29	28.5	28.7	28.8	29
MCHC (g/L)	320-360	31.5 (L)	31.6 (L)	31.9 (L)	31.9 (L)	32.5
Platelet Count (×10^9^/L)	150-450	112 (L)	94 (L)	52 (L)	65 (L)	77 (L)
MPV	9.4-12.3	9.8 (L)	10.1 (L)	10.4 (L)	11	10.5
Neutrophil Rel %	40-70	61.6	77.8 (H)	57.9	66.3	60.4
Lymphocyte Rel %	20-40	26.8	13.4 (L)	30.9	22.4	29
Monocyte Rel %	2-8	9.8	7.3	8.7	9.3	8.50
Eosinophil Rel %	1-4	1.3	1	1.9	1.3	1.4
Basophil Rel %	0-1	0.2	0.1	0.3	0.3	0.1
Immature Granulocytes Rel %	0-0.4	0.3	0.4	0.3	0.4	0.6 (H)
Retic Relative %	0.5-2.5	1.80 (H)	-	-	-	-
Retic Absolute (10⁹/L)	0.02-0.08	0.059	-	-	-	-
Glucose (mmol/L)	70-99	126 (H)	104	156 (H)	172 (H)	146 (H)
HbA1c	<5.7 normal, >6.5 diabetes	-	-	6.7 (H)	-	-
Sodium (mmol/L)	135-145	142	140	136	140	139
Potassium (mmol/L)	3.5-5.0	3.9	5	3.9	3.9	3.6
Chloride (mmol/L)	98-106	103	103	102	104	104
CO2	23-29	32 (H)	28	30	30	28
Calcium (mmol/L)	8.5-10.5	8.3 (L)	9.1	7.8 (L)	8.6	8.1 (L)
BUN	7-20	36 (H)	26 (H)	39 (H)	26 (H)	26 (H)
Creatinine (µmol/L)	0.6-1.3	2.43 (H)	2.04 (H)	2.13 (H)	1.84 (H)	1.69 (H)
Albumin (g/L)	3.5-5.0	3.3 (L)	4.0	3.1 (L)	-	-
ALP	44-147	45	53	45	-	-
ALT (U/L)	7-56	13	19	15	-	-
AST (U/L)	10-40	8 (L)	29	9 (L)	-	-
Lipase level	0-160	-	38	-	-	-
eGFR CKD-EPI	>60	23 (L)	29 (L)	27 (L)	32 (L)	36 (L)
Total Bilirubin (µmol/L)	0.1-1.2	0.40	0.6	0.4	-	-
Magnesium (mmol/L)	1.7-2.2	-	2.3	2.0	1.9	1.8 (L)
Phosphate (mmol/L)	2.5-4.5	-	3.5	3.0	3.1	3.0
Total Protein	6.0-8.3	5.4 (L)	6.9	5.1 (L)	-	-
Total Cholesterol (mg/dL)	<200	-	-	152	-	-
HDL (mg/dL)	>40 (M); >50 (F)	-	-	35	-	-
LDL (mg/dL)	<100	-	-	43	-	-
Triglycerides (mg/dL)	<150	-	-	369 (H)	-	-
TSH	0.4-4.0	-	-	2.04	-	-

The initial differential diagnoses included the following: chronic pancreatitis, chronic dyspepsia, medication-induced colitis, gastritis, and constipation. However, to identify the root cause of this patient's pancreatitis, the healthcare team decided to perform a thorough review of her medical history. Her gastrointestinal history was significant for chronic and acute-on-chronic gastritis, complicated colitis, and a hiatal hernia. One month prior, she had undergone a workup with multiple esophagogastroduodenoscopies (EGDs), colonoscopies, and a gastric emptying test, which yielded negative results. 

Further evaluation with abdominal ultrasound revealed a heterogeneous, prominent pancreas; perihepatic fluid; and left perinephric fluid. Autoimmune serologies, including anti-saccharomyces cerevisiae antibodies (IgA, IgG), endomysial IgA, and gliadin IgA/IgG, were within normal limits. An EGD performed during hospitalization showed only mild gastritis of the gastric body and antrum, insufficient to explain her symptoms. Thereafter, a medication-related etiology was suspected, resulting in a holistic review of her medications. At the time of her admission, her medication regimen consisted of the following: mesalamine (800 mg PO two times daily); apixaban (Eliquis) (5 mg PO two times daily); atorvastatin (40 mg PO two times daily); carvedilol (25 mg PO two times daily); famotidine (20 mg PO once daily); hydralazine (50 mg PO three times daily); levothyroxine (100 mcg PO once daily); multivitamin Rena-Vite Rx (PO once daily); sucralfate (1 g PO two times daily); and ferrous sulfate (325 mg PO once daily). Mesalamine was identified as a potential culprit given its documented association with DIP [8]. As a result, mesalamine was discontinued during this admission. The mesalamine manufacturer details and batch/lot number were not available, as the medication was provided in a pill organizer without original packaging. 

Management of her CKD focused on avoiding nephrotoxins and adjusting medications to renal function, such as reducing the Eliquis dosage to 2.5 mg two times daily. Her congestive heart failure, hypertension, age ≥75 years, diabetes mellitus, stroke/transient ischemic attack - vascular disease, age 65-74 years, sex category (CHA₂DS₂-VASc) score was computed at three, and her hypertension, abnormal renal/liver function, stroke, bleeding history or predisposition, labile INR, elderly, drugs/alcohol concomitantly (HAS-BLED) score was computed at 1. Due to this, she was managed with chronic anticoagulation. For the patient’s type 2 diabetes mellitus, mild proteinuria, and recent HbA1c of 6.9% at her prior hospitalization (10/6/2024), managed with insulin glargine 15 units subcutaneously at bedtime and dietary modifications. She was counseled on lifestyle and dietary changes to manage her diabetes and committed to adherence. She received metoclopramide 10 mg PO PRN and hydromorphone (Dilaudid) 0.2 mg (0.2 mL) injection IV push PRN for nausea and pain, respectively. 

Following discontinuation of mesalamine, the patient’s abdominal pain and nausea improved significantly. She remained hemodynamically stable and was discharged on hospital day 6 with recommendations for gastroenterology follow-up. Discharge instructions, diagnostic findings, and the care plan were discussed with the patient, who demonstrated understanding and agreement. Institutional ethics approval was obtained for publication of this case report, and written informed consent was secured from the patient for publication of her clinical details and accompanying images.

## Discussion

DIP is a rare etiology of AP [4]. The variability in reported prevalence stems from differences in population demographics, diagnostic criteria, and underreporting [2]. Studies estimate the incidence of drug-induced AP to be approximately 3.6% [3], with several population-based studies citing a prevalence closer to 2% [4,9,10]. DIP presents similarly to other forms of AP, with symptoms including epigastric pain radiating to the back, nausea, and vomiting [1]. In more severe cases, patients may exhibit jaundice, systemic symptoms such as fever and hypotension, or develop complications like pseudocysts or sepsis [1]. Altered mental status may also occur due to the toxic effects of the causative medication or concurrent systemic illness [4]. Most serious complications tend to occur within the first 48 hours, underscoring the importance of early recognition and appropriate management [1].

The diagnosis of DIP remains inherently challenging and is primarily one of exclusion. In our patient, gallstones were excluded by abdominal ultrasound, autoimmune pancreatitis by negative serologies, and structural gastrointestinal causes by prior negative endoscopic evaluations. Laboratory findings demonstrated triglycerides below the threshold typically associated with pancreatitis and no hypercalcemia, further excluding metabolic causes. These findings collectively supported a drug-induced etiology, with mesalamine identified as the most likely culprit [8-11]. In the context of our patient’s presentation, her symptoms and clinical history were consistent with AP. Despite normal serum lipase levels, the constellation of persistent abdominal pain, CT imaging findings suggestive of pancreatitis, and the exclusion of other common causes supported the diagnosis. The patient’s polypharmacy, particularly the use of mesalamine, a drug with a documented association with DIP, warranted careful evaluation [8]. Discontinuation of mesalamine led to symptom resolution, further reinforcing the diagnosis of DIP in this case. For pharmacovigilance and adverse drug reaction (ADR) reporting, documentation of the drug’s brand name, manufacturer, and batch/lot number is highly beneficial. Although this information was unavailable in our case, we emphasize the importance of collecting it in clinical practice to strengthen ADR reporting and improve post-marketing safety surveillance.

To assess causality, a systematic review of the patient’s medications was conducted. Other agents in her regimen, including apixaban, carvedilol, hydralazine, atorvastatin, levothyroxine, famotidine, sucralfate, ferrous sulfate, and multivitamins, have been associated with pancreatitis only rarely or not at all, with evidence largely limited to isolated case reports or insufficient pharmacovigilance data [11]. In contrast, mesalamine has a well-documented association with DIP [12]. According to the World Health Organization-Uppsala Monitoring Centre (WHO-UMC) causality assessment, the temporal relationship between mesalamine use and symptom onset, the resolution of symptoms following drug withdrawal (positive dechallenge), and the exclusion of more common etiologies support a classification of probable/likely DIP in this case [13]. A rechallenge was not attempted, in line with ethical considerations.

Our patient had been on chronic mesalamine therapy for inflammatory bowel disease, and her presentation is consistent with previously published mesalamine-induced pancreatitis cases, in which symptom onset has ranged from days to months after initiation and improvement followed discontinuation [8,12]. Similar to other reported cases, our patient developed AP during mesalamine therapy and improved after drug withdrawal. However, unlike many prior reports where elevated lipase was present, our patient had normal serum lipase, a phenomenon that has been described in rare instances of AP confirmed by imaging [14]. Although elevated lipase is a sensitive and specific marker, diagnosis per the Revised Atlanta Classification remains valid with two of three criteria (clinical pain, imaging, enzyme elevation), even when enzymes are normal [15]. Her underlying CKD and transient acute kidney injury may have contributed to this atypical laboratory profile, as impaired renal clearance can influence enzyme kinetics [14]. Notably, the patient's creatinine improved from 2.5 mg/dL on admission to 1.7 mg/dL at discharge, further contextualizing her presentation. This underscores the importance of imaging and clinical suspicion in diagnosing pancreatitis with atypical laboratory findings.

## Conclusions

This case highlights the diagnostic challenges of DIP, particularly in patients with complex comorbidities and polypharmacy. A thorough review of the patient’s comorbidities and medications was essential in identifying mesalamine as the likely offending agent. Prior diagnostic studies, including multiple endoscopies and a gastric emptying study, aided in ruling out more common causes of AP such as gallstones, alcohol use, and hypertriglyceridemia. This underscores the importance of maintaining a broad differential and thoroughly evaluating medication history when faced with idiopathic pancreatitis.

DIP remains a diagnosis of exclusion, and clinician awareness of less common causes like mesalamine is vital, especially in patients with extensive drug regimens. Imaging, laboratory workup, and careful medication review are critical in establishing the diagnosis. Our case further highlights that normal lipase values may occur in AP, particularly in the setting of renal dysfunction, reinforcing the need for clinical vigilance and reliance on imaging when laboratory findings are inconclusive. In this case, symptom resolution following the withdrawal of mesalamine supports its role as the causative agent. Early recognition and discontinuation of the offending drug, along with supportive care, remain the cornerstone of DIP management and are essential for preventing complications and improving patient outcomes.
